# Mental and physiological wellbeing while rowing across the North Atlantic: a single-case study of subjective versus objective data

**DOI:** 10.3389/fphys.2023.1244438

**Published:** 2023-09-19

**Authors:** Klaus Zeiner, Babak Dabiri, Ciara Burns, Lena Kummer, Eugenijus Kaniusas

**Affiliations:** Institute of Biomedical Electronics, TU Wien, Vienna, Austria

**Keywords:** unassisted rowing, resilience, autonomic nervous system, sleep, heart rate variability, circadian cycle

## Abstract

**Introduction:** Unassisted rowing across the Atlantic Ocean is an extreme undertaking challenging the human body in every possible way. The reported rowing journey lasted for 42 days in a small vessel with 12 rowers, each rowing for 12 h a day, broken into 3 h shifts. This schedule disrupts the natural circadian cycle and autonomic balance, affecting subjective and objective wellbeing and sleep quality, that lack continuous empirical quantification.

**Methods:** With a self-reported questionnaire and objective heart rate variability measurements every second day in a single female rower, we monitor evolutions of the subjective sleep quality and mental wellbeing as well as autonomic body control over the journey duration. We evaluate the hypothesis that extreme rowing impairs subjective and objective data in a similar way over time and that 3 h shifts diminish the circadian rhythm of the autonomic body control.

**Results:** The sleep quality was mainly influenced by wake ups during sleep, while mental wellbeing was predominantly influenced by physical exhaustion. The perceived sleep quality and wellbeing dropped 2–3 days after the start with the rower not yet accommodated, in the middle of the journey with major wake ups, and again 5–6 days prior to the end with major exhaustion of the participant. Evolutions of the subjective perceptions diverge from that of the heart rate variability. The body’s autonomic recovery during short sleep periods progressively decreases over the journey duration while the vagal activity rises and the sympathovagal balance shifts towards vagal tone. The shifts of 3 h weaken the circadian rhythm of the heart rate variability.

**Discussion:** Our results demonstrate how human body meets extreme mental and physical exhaustion on the high seas. The gained physiological and psychological insights also offer a basis for effective preparation of undertakings involving extreme physical exhaustion and sleep deprivation.

## 1 Introduction

Rowing, which targets both strength and endurance, is acknowledged to improve health and comprehensive wellbeing. This physical activity aids in reducing hypertension, the risk of developing diabetes, and heart disease (WHO, 2009). In particular, rowing improves cardiorespiratory fitness while mitigating aging-related pathologies such as sarcopenia or osteoporosis (Volianitis et al., 2020). These positive effects have been shown for moderate levels of physical activity, e.g., improved autonomic balance with increased parasympathetic modulation (Aubert et al., 2003), activity lasting at least 30 min each day to improve and maintain health (Pate et al., 1995). Large or extreme levels of activity, however, raise the risk of negative consequences such as injuries, since muscles, tendons and joints are highly stressed and overused in permanent attempts to improve or deliver physical capacity.

Unassisted rowing across the Atlantic Ocean is an extreme undertaking. It includes not only physical stressors (incl. physical fatigue, injuries, sleep deprivation with disrupted homeostasis, metabolic stress, limited food and clothing, sea sickness) but also psychological stressors (mental fatigue, isolation, sensory deprivation), environmental stressors (threat of hitting floating containers, difficult environmental conditions), technical stressors (boat problems, water desalination), organizational stressors (hectic schedule, COVID-19 restrictions), and personal stressors (limited communication with family) (Weston et al., 2009); (Keohane et al., 2019).

Thus, mental and physiological factors and their interrelations [e.g., via coping strategies (Alschuler et al., 2020)] are pivotal to the success of transatlantic rowing. Moreover, these factors are highly instructive from a psychological and physiological perspective when humans face adversities (Alschuler et al., 2019). As reported in (Keohane et al., 2019), comprehensive physiological testing of cardiorespiratory and immunological function was conducted before and after the completion of transatlantic rowing but not throughout it. A subjective questionnaire-based assessment of physical and psychological experiences was also reported during transatlantic rowing (Alschuler et al., 2020), showing a decrease in the restfulness and oscillating positive emotions over the journey duration; however, no concurrent objective data were obtained. Sleep deprivation which occurs during most of the extreme events and poses a true physiological and psychological challenge, is currently subject to research limited to self-reports (Weston et al., 2009); (Graham et al., 2012). In particular, sleep deprivation and fragmented sleep increase energy expenditure of the body, which increases daily energy requirements (Jung et al., 2011). Consequently, objective and continuously recorded data are required on psychological and physiological functions during extreme activities, which would advance the knowledge on the human excellence under adverse conditions.

The present study presents the first investigation of a long-duration exhausting activity on the high sea under difficult environmental conditions based on a case study using periodic subjective self-reports and objective self-measurements (mixed-method design). The self-reports assess the sleep quality and mental wellbeing [defined as a state in which the individual can cope with stresses of life (Cresswell-Smith et al., 2022)]. The self-measurements reflect the autonomic body control, specifically, the ability of the body to adapt to varying physiological, psychological, and environmental challenges. This makes the autonomic body control a valuable indicator of both sleep quality (Fink et al., 2018) and mental wellbeing (Kemp and Quintana, 2013).

The main hypothesis is that the extreme transatlantic rowing of a fitness enthusiast results in a decline in the sleep quality, mental wellbeing, and in the modulation capacity of the autonomic body control over the course of the journey. Additional hypotheses are that the rowing similarly deteriorates both the subjective perception (of sleep and wellbeing) and objective measurements (of the autonomic body control) over time; as well as that periodic 3 h shifts of rowing diminish the 24 h circadian rhythm of the autonomic body control.

## 2 Materials and methods

### 2.1 Data collection

**FIGURE 1:**
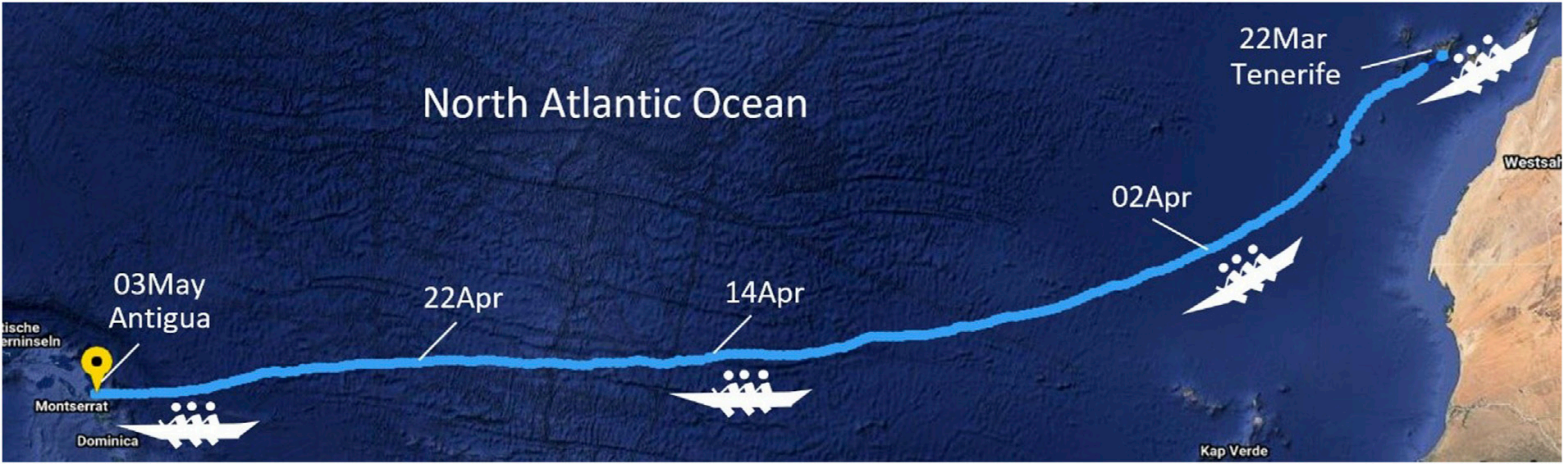
Rowing across the North Atlantic—the course of the journey.

The data used for this study was collected in a prospective, longitudinal, single case study during an east-to-west row of the North Atlantic Ocean (Figure 1). The row started on 22 March 2021 11a.m. in Tenerife and finished 42 days later on 3 May 2021 1p.m. in Antigua, covering the total travelled distance of 6,185 km (from GPS tracker). As a member of a 12 person rowing team, the subject was a woman in her mid 20 s (67 kg at the start, height 1.64 m), with no prior ocean rowing experience. She had prior experience in team sport, but none in any ultra-endurance competition. The expedition was conducted in a relatively small vessel especially designed for ocean crossings (Roxy Atlantic 2021), with six rowing positions and just enough space to rest for the other six persons.

Ethical guidelines were implemented including signed detailed information letter and signed declaration of consent of the participant. The study was co-designed, guided, and reviewed by the Pilot Research Ethics Committee of TU Wien (March, 2021).

Journey data such as boat position, speed, and local temperature were continuously tracked with the on-board navigation system. During the whole journey, a 3 h rhythm of rowing and resting was kept; see Figure 4 for exact daytime of the subsequent rowing and resting phases (given in UTC+2). The resting phases were mostly used for eating, sleeping, and personal care, so that the effective sleep phases were shorter than 3 h and the awake phases longer than 3 h (Figure 6).

**FIGURE 2:**
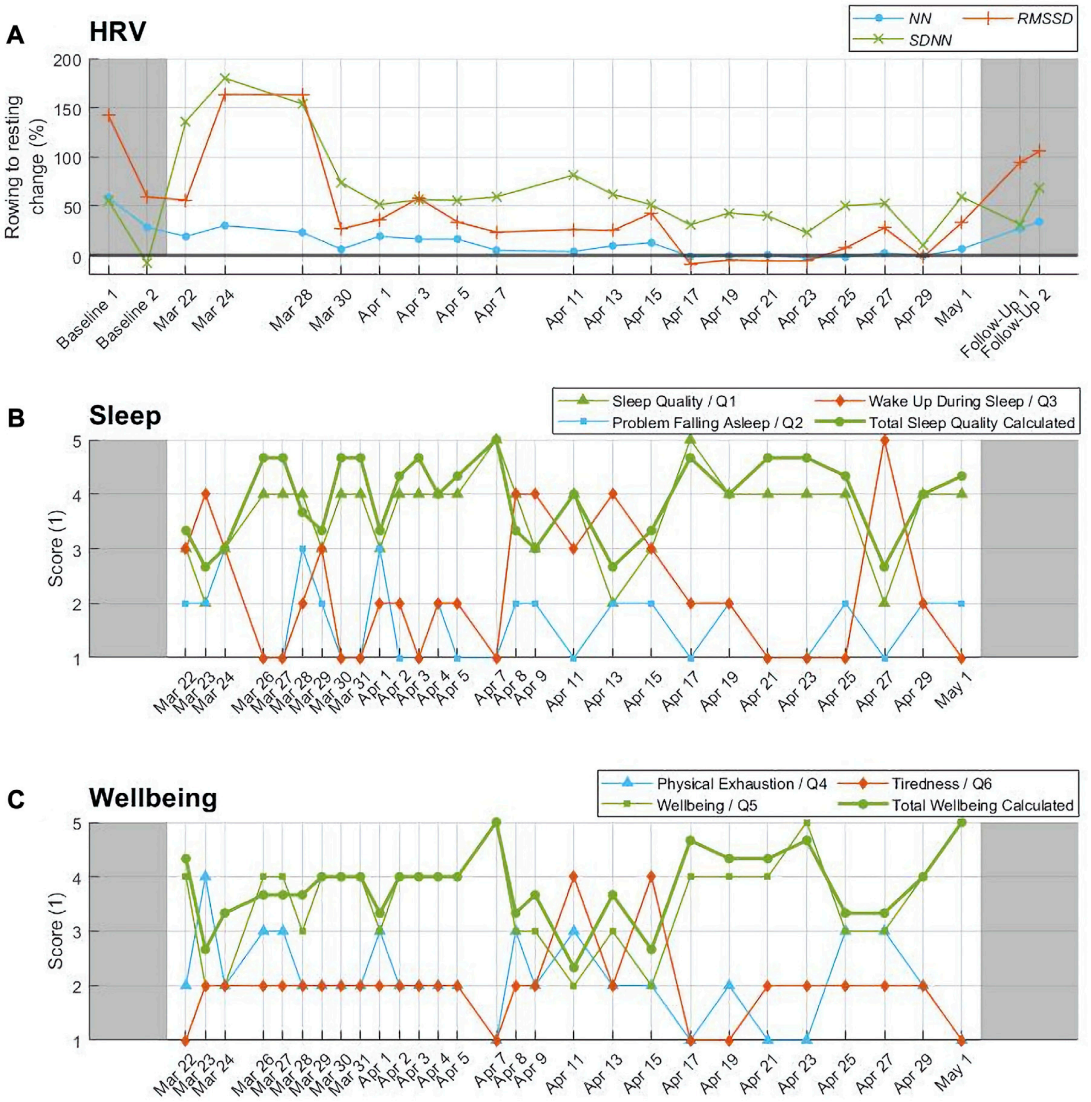
Objective HRV parameters versus subjective questionnaire reports. Time courses of **(A)** the percentage change in the medians of HRV parameters *NN*, *SDNN*, and *RMSSD* from rowing to resting phases, **(B)** subjective self-reported sleep quality, and **(C)** subjective self-reported mental wellbeing.

Electrocardiography (ECG) measurements were performed with a waterproof one channel Holter ECG monitor at a sampling rate of 1,000 Hz (Bittium Faros, Bittium Corporation, Finland). The first baseline ECG recording (Baseline 1 in Figure 2A) spanned 24 h and took 49 days before the journey, including 3 consecutive cycles of 2 h rowing and 2 h resting in the morning hours of that day. The second baseline ECG recording (Baseline 2 in Figure 2A) was carried out for 24 h 2 days before the journey; the recording did not include any rowing activity but rather stressful journey preparations, with still a rather normal sleep wake rhythm. During the journey, recordings were performed every second day for 24 h (Figure 2A). Adhesive electrode positions on the chest were varied in that they were placed in the middle of the thorax or in the area below the claviculae. This was necessary to keep skin’s irritations at a minimum, as caused by adhesives exposed to continuous sweat and oceanic salt. The recorded (and accumulated) data remained stored on the ECG monitor during the whole journey and was uploaded to a PC only after the end of the transatlantic crossing. In addition, 10 and 11 days after the crossing, two more follow-up ECG recordings (Follow-Up 1 and 2 in Figure 2A) were performed, without any rowing activity.

Right after each 24 h of ECG recording, the participant filled a self-reported proprietary questionnaire on a smartphone, including 3 items about the sleep quality during the prior 24 h:

- Question Q1 “How do you rate your sleep quality in the last 24 h?,”
- Q2 “Did you have problems falling asleep during sleep periods in the last 24 h?”, and
- Q3 “Did you wake up during sleep periods in the last 24 h?”

and 3 more items about the physical exhaustion and mental wellbeing:

- Q4 “Was it physically exhausting to row in the last 24 h?”,
- Q5 “How do you rate your wellbeing in the last 24 h?” and
- Q6 “Did you feel tired during daytime rowing in the last 24 h?”

Each item had to be rated with the score *S* between 1 (very poor, no problems, no wake ups, very easy, very low, not at all for Q1-Q6, respectively) and 5 (very good, problems only, many wake ups, very exhausting, very high, very often) depending on how accurate the item described the prior 24 h. In addition, an average score for the total sleep quality (average of all three *S* of Q1, -*S* of Q2, and -*S* of Q3, plus four) and another average score for the total wellbeing (average of -*S* of Q4, *S* of Q5, and -*S* of Q6, plus four) were calculated. The higher the average score, the better the sleep quality and mental wellbeing, respectively. Positively and negatively worded questions composed this questionnaire to mitigate its potential bias as compared to only positively or negatively worded questions (Murphy et al., 1989). An option of a brief diary was provided on the smartphone every second day to note potential adversities and health issues, to be filled together with the questionnaire.

### 2.2 Data analysis

ECG data were manually checked by a trained person for consistency and then analyzed with Bittium Cardiac Monitor (Bittium Corporation, Finland). First, the overall quality of the individual 24 h recordings was checked: if the recording included more than 40% of valid ECG data, it was considered for further analysis. Then R peaks of ECG were detected. All detected R peaks and the associated ECG waveform were validated in windows of 2–3 min by the trained person. If still a low ECG quality (noise, movement artefacts) or false detected R peaks (including ectopic beats) were found, they were marked as artefacts. All 5 min windows with more than 50% artefact-free R peaks and ECG data were included after this manual check.

The heart rate (HR) and the standard parameters of the heart rate variability (HRV) (Malik et al., 1996)—to assess changes in the autonomic nervous system modulated by the vagus nerve of the heart - were calculated for the consecutive non-overlapping 5 min intervals of the included data. These parameters are the normal-to-normal (NN) heart beat interval *NN*, the standard deviation *SDNN* of the NN intervals as a measure of the overall HRV considering both sympathetic and parasympathetic activities, the root-mean-square of the differences of consecutive NN intervals *RMSSD* as a measure of the fast parasympathetic activity, the power of the high frequency *HF* components (0.14–0.4 Hz) of the NN signal as a measure of the respiration-related parasympathetic activity, and the ratio *LF/HF* of the power of low frequency *LF* components (0.04–0.14 Hz) to *HF* as a measure of the sympathovagal balance.

Further analysis was performed in Matlab (R2021a, The MathWorks Inc.). A relative change of HRV parameters from rowing to resting phases was calculated for each 24 h measurement, as a measure of the body’s recovery efficiency during resting phases (Figure 2A). Here the percentage quotient of the median of all 5 min interval values during resting to the median of all 5 min interval values during rowing was calculated. In order to compare the objective HRV data with the subjective questionnaire-related data, the timelines of the latter quotients were compared to the timelines of the sleep quality and mental wellbeing scores.

**FIGURE 3:**
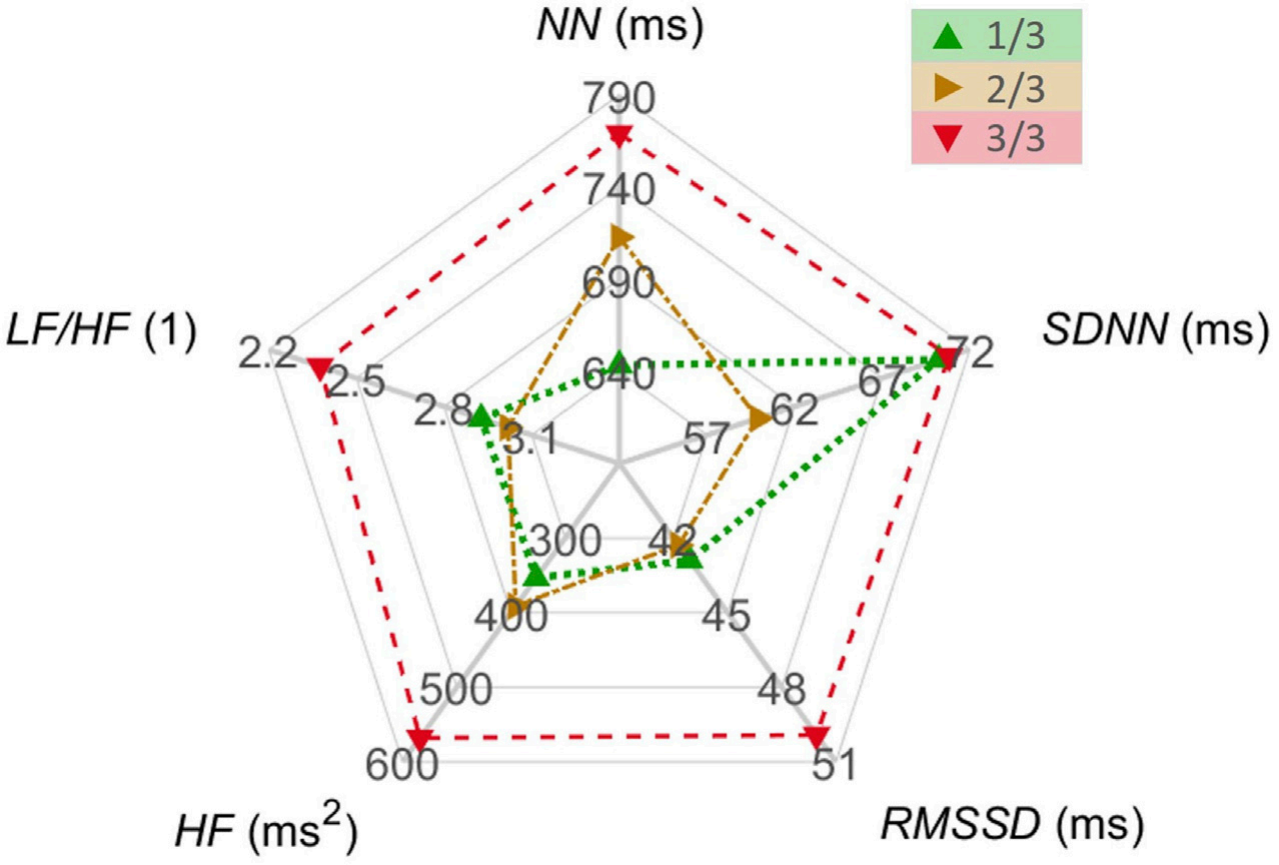
HRV parameters *NN*, *SDNN*, *RMSSD*, *HF*, and *LF/HF* within the first, second, and the final third of the journey, coded with green, yellow and red colors, respectively. The medians of the medians of the 5 min interval values of the respective parameter are shown.

**FIGURE 4:**
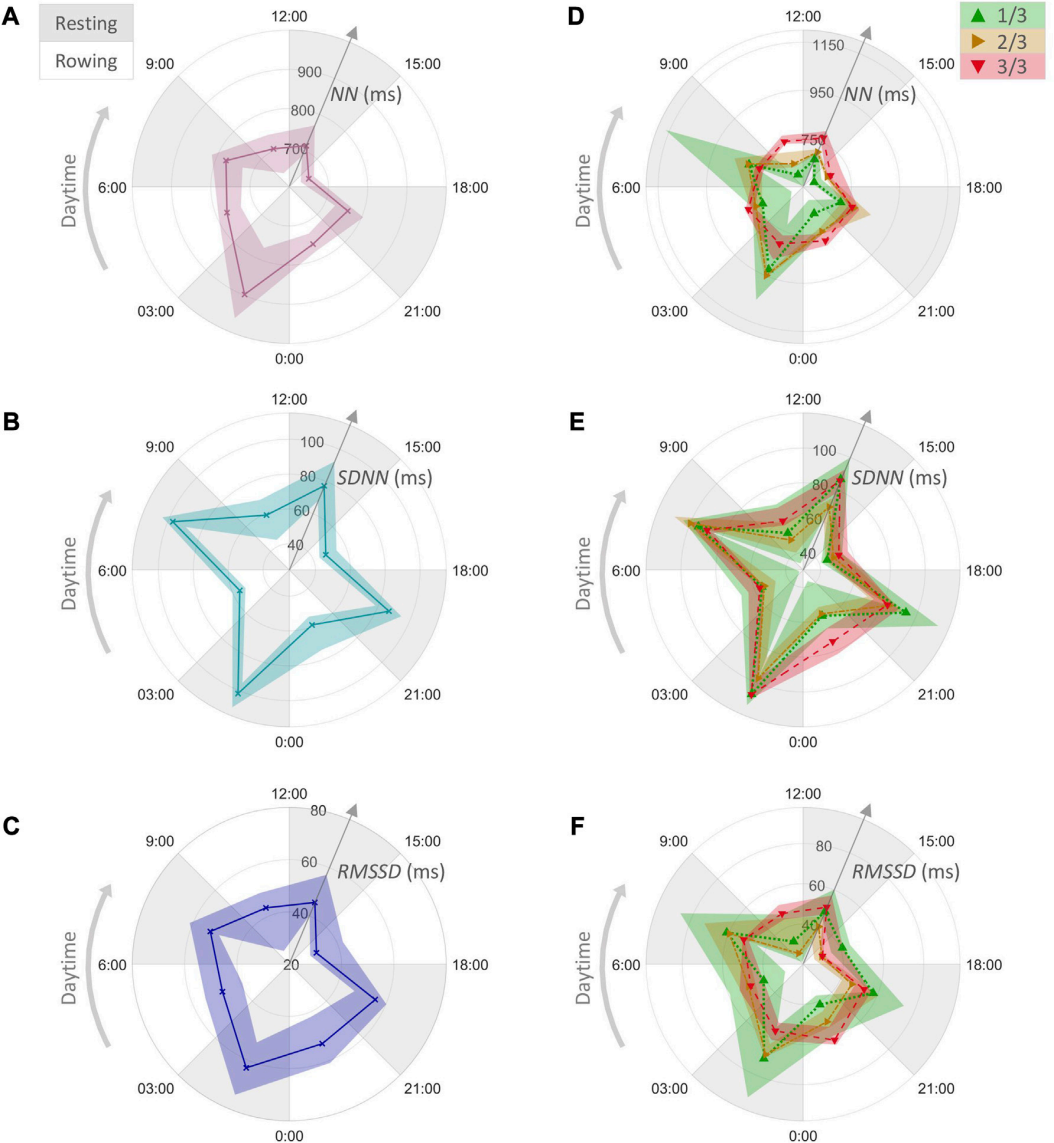
HRV parameters for rowing phases (white sectors) and resting phases (grey sectors) over 24 h. The median and interquartile range (25% and 75%) are shown for the medians of the 5 min interval values within each phase. **(A)** The values of *NN*, **(B)** *SDNN*, and **(C)** *RMSSD* for the whole journey. **(D)** The values of *NN*, **(E)** *SDNN*, and **(F)** *RMSSD* within the first, second, and the final third of the journey, coded with green, yellow and red colors, respectively.

**FIGURE 5:**
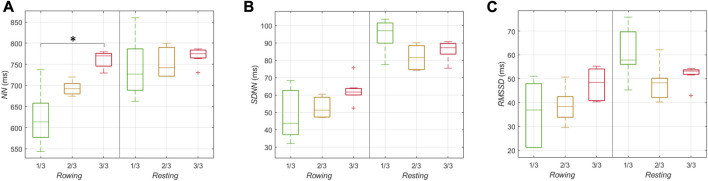
HRV parameters for 3 h rowing phases and 3 h resting phases within the first, second, and the final third of the journey, coded with green, yellow and red colors, respectively. **(A)** The distribution of quartiles of the 5 min interval values of *NN*, **(B)** *SDNN*, and **(C)** *RMSSD*. The statistical difference is indicated by asterisk “*” with the error probability *p* < 0.01. Dashed lines indicate whiskers extending to the most extreme data points.

**FIGURE 6:**
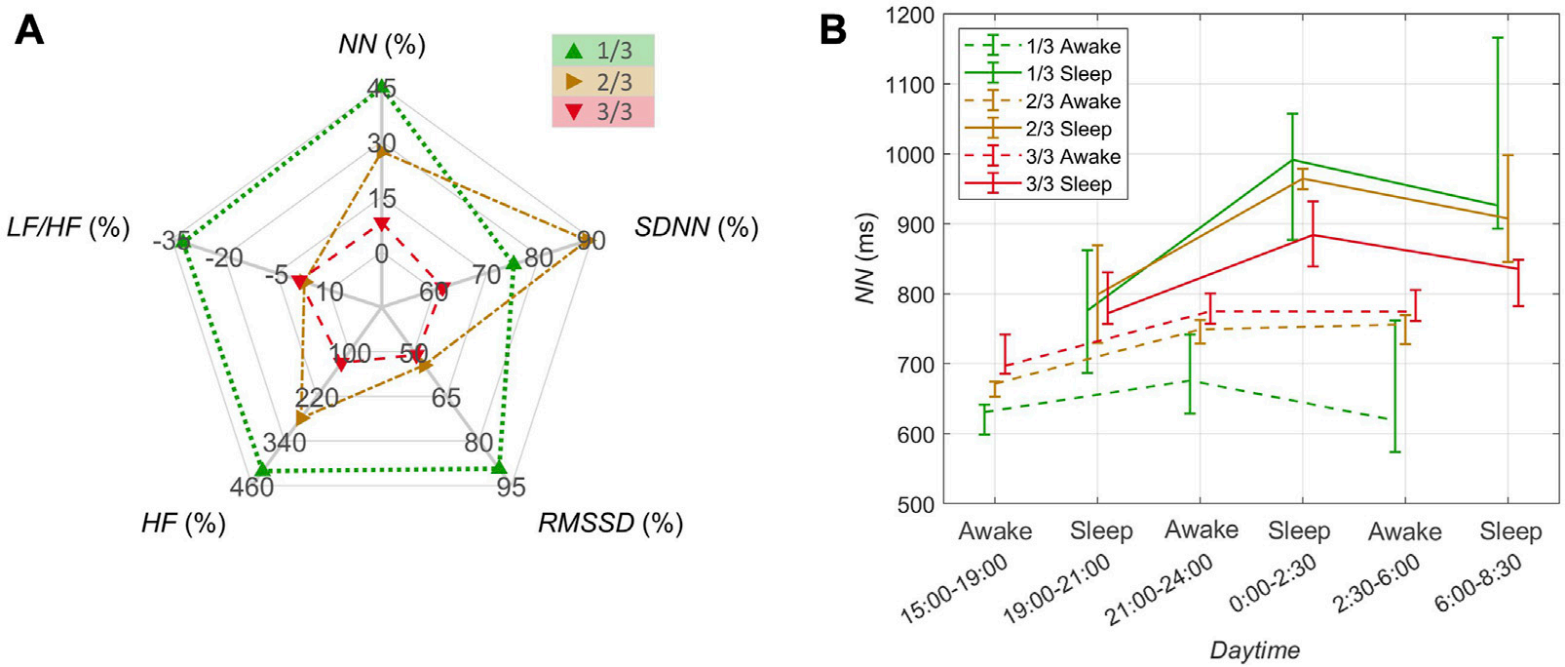
HRV parameters for <3 h sleep phases and >3 h awake phases within the first, second, and the final third of the journey, coded with green, yellow and red colors, respectively. **(A)** The percentage change in the median of the medians of the 5 min interval values of *NN*, *SDNN*, *RMSSD*, *HF*, and *LF/HF* from awake to sleep phases. **(B)** Time-resolved changes in *NN*. The distribution of quartiles of the 5 min interval values are shown along the daytime for subsequent sleep phases (solid lines) and awake phases (dashed lines).

The alternating resting and rowing phases were considered in HRV parameters in that the medians of the 5 min interval values of each separate phase were calculated. The distribution of these phase-specific medians was then calculated for the first third (March 22 to April 3), the second third (April 5 to April 17), and the final third (April 19 to May 1) of the total duration of the journey (Figures 3–5). The same procedure was applied to analysis of awake phases (including rowing and awake resting with >3 h in total) and sleep phases (with <3 h) (Figure 6).

**FIGURE 7:**
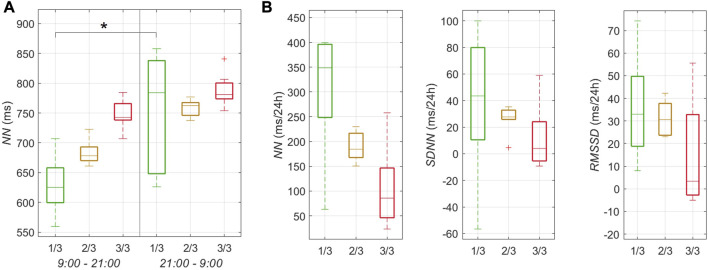
Circadian rhythms of HRV parameters within the first, second, and the final third of the journey, coded with green, yellow and red colors, respectively. **(A)** The distribution of quartiles of the medians of the 5 min interval *NN* values for 12 h phases (9:00–21:00 and 21:00–9:00). **(B)** The distribution of quartiles of the change of *NN*, *SDNN*, and *RMSSD* over 24 h (9:00–9:00) from the linear regression analysis. The statistical difference is indicated by asterisk “*” with the error probability *p* < 0.01. Dashed lines indicate whiskers extending to the most extreme data points.

In order to evaluate the circadian rhythm, the distribution of NN intervals from 9:00 to 21:00 as well as from 21:00 to 9:00 was calculated for each third of the journey (Figure 7). Additionally, the absolute change in *NN, SDNN,* and *RMSSD* over 24 h period (9:00 to 9:00) was calculated via linear regression analysis.

Rowing and resting NN intervals of all thirds as well as NN intervals grouped in the two 12 h blocks were compared using ANOVA in Matlab (R2021a, The MathWorks Inc.), with the level of significance at *p* < 0.01.

## 3 Results

### 3.1 Rower, boat, and ambient data

The subject took medication (Biodramine caffeine, with dimenhydrilate as the active substance) every 6 h against seasickness, beginning 24 h before the journey start and continuing for 10 days. In addition, she applied a transdermal plaster (Scopoderm TTS, with Scopolamin as the active substance) behind the ear for the first 7 days of the journey to mitigate seasickness. The menstrual cycle ceased with no bleeding at all during the journey so that two cycles were missed in total. The subject’s weight was 62 kg at the end of the journey. The optional brief diary was not used, most likely due to an intense schedule on the boat.

The average speed of the boat was 4.7 ± 1.57 km/h (or 2.54 ± 0.85 knot) with the average logged distance 22.96 ± 12.59 km travelled per 4 h. The outside temperature rose during the journey, i.e., 21.68°C ± 3.76°C (with the circadian day to night variation 7.3°C ± 3.14°C) in the first third of the journey, 24.06°C ± 2.85°C (6.78°C ± 1.42°C) and 26.53°C ± 2.45°C (5.72°C ± 1.4°C) in the second and last thirds, respectively. See Supplementary Figure S1 for details.

### 3.2 ECG quality check

The initial quality check excluded about 10% of ECG data from the analysis due to a low ECG quality. From the remaining data, 26% of R peaks were excluded after their manual validation. In total, 19 24 h recordings from the high sea were included and 1 excluded (Figure 2A).

### 3.3 Subjective questionnaire reports

The temporal evolution of self-reported scores on individual sleep-related questions Q1-3 (Figure 2B) illustrated a relatively high sleep quality (Q1) with the average of 3.64 ± 0.78, little problems falling asleep (Q2) with only 1.64 ± 0.67, and significant wake ups (Q3) with 2.21 ± 1.19. While there were three temporal drops in the perceived sleep quality in the first few days of the journey (March 22–24), in the middle of the journey (April 9–15), and close to the end of it (April 27), the problems falling asleep peaked semi-periodically only in the first third of the journey, and the frequency of wake ups behaved inversely to that of the sleep quality (mutual correlation of −0.79). The average score for the total sleep quality resembled that of the sleep quality (Q1), with an obvious oscillation of about 4 days in the first half of the journey (Figure 2B).

The temporal evolution of self-reported scores on individual wellbeing-related questions Q4-6 (Figure 2C) illustrated medium values of exhaustion (Q4) with 2.14 ± 0.76, wellbeing (Q5) 3.57 ± 0.87, and tiredness (Q6) 1.96 ± 0.69. The physical exhaustion peaked at the beginning, in the middle, and at the end of the crossing. The wellbeing seemed to be inversely related to exhaustion (correlation of −0.87), with almost continuously perceived slight tiredness which accumulated significantly in the middle of the journey. The average score for the total wellbeing followed closely that of the wellbeing (Q5).

### 3.4 Objective HRV data

The relative change of *NN, SDNN*, and *RMSSD* from rowing to resting are shown in Figure 2A; namely, from the Baselines 1–2 (i.e., the adaptation from active daytime to resting sleep), over the journey duration, down to the Follow-ups 1–2 (i.e., the adaptation from active daytime to resting sleep). The Baseline 1 value of *NN* of about 60% decreased to about 20% (March 22) at the start of the journey and then increased again to about 30% (March 24) within the first third of the journey. Afterwards, the relative change in *NN* decreased throughout the row down to about 0% (no adaptation) in the last third, followed by a recovery to about 40% in the Follow-ups 1–2. The values of *SDNN* and *RMSSD* decreased similarly throughout the journey; however, peak values within the first third (180% for *SDNN* and 160% for *RMSSD*, March 24) clearly surpassed the values in the Baselines 1-2 and Follow-ups 1–2 (max. of 60% for *SDNN* and max. 140% for *RMSSD*).

Figure 3 illustrates the medians of all HRV parameters *NN, SDNN, RMSSD, HF*, and *LF/HF* differentiated for the individual thirds of the journey. The *NN* median increased by about 20% from 640 ms in the first third to 770 ms in the last third of the journey. The *SDNN* median started at a level of 70 ms, decreased by about 14% to 60 ms in the second third, and then recovered back to 70 ms in the last third. The medians of *RMSSD, HF*, and *LF/HF* did not change from the first to the second third but then *RMSSD* and *HF* increased and *LF/HF* decreased in the final third. Specifically, *RMSSD* increased by about 16% from 43 ms in the first third to 50 ms in the last third, *HF* increased by about 66% from 350 ms^2^ to 580 ms^2^, and *LF/HF* decreased by about 19% from 2.9 to 2.35.

Figure 4A shows the distribution of *NN* within phases of rowing and resting over 24 h for the whole journey, while Figure 4D shows this distribution split into the thirds of the journey. During the first third, *NN* values clearly increased during resting as compared to rowing; however, these *NN* changes weakened in the second third and almost disappeared in the last third (for details see Figure 5A). Increased *NN* values during the night time (0:00–6:00) in the first third pointed to the presence of the circadian 24 h rhythm, with this difference also disappearing in the last third (Figures 4A, D). During resting phases, the values of *SDNN* (Figures 4B, E) and *RMSSD* (Figures 4C, F) also increased in comparison to rowing, with these differences being more pronounced in the first third of the journey (Figures 4E, F) and less pronounced for *RMSSD* (Figures 4B, C). The values of *HF* increased similarly while the ratio *LF*/*HF* decreased during resting phases, especially in the first third of the journey (see Supplementary Figure S2).

Detailed changes in *NN* within resting and rowing phases (3 h each) are summarized in Figure 5A. The median *NN* significantly raised during rowing phases from the first to the last third of the journey; there was a similar tendency of increase during resting phases but statistically insignificant. Figures 5B, C depict the associated changes of *SDNN* and *RMSSD*, showing a tendency of *SDNN* and *RMSSD* to rise from the first to the last third while rowing. Similarly, there was a tendency of *HF* to increase during rowing, with no clear tendencies observed in the ratio *LF*/*HF* (see Supplementary Figure S3).

Figure 6A depicts the relative change in the medians of *NN, SDNN, RMSSD, HF*, and *LF/HF* from awake phases (>3 h) to sleep phases (<3 h) for the individual thirds. In analogy with the qualitative *NN* observation from Figure 4D, these changes—as directly related to the body’s recovery—in *NN, RMSSD*, and *HF* were highest for the first third of the journey and lowest for the last third; with the exception of *SDNN*, its changes peaked in the middle of the journey. In contrast, the change in the ratio *LF/HF* decreased as the journey progressed.

Detailed changes in *NN* within awake and sleep phases are summarized in Figure 6B. While the median *NN* during awake phases increased over the course of the journey, the median *NN* during sleep phases decreased. Correspondingly, the difference in *NN* between sleep and awake phases decreased as the journey time progressed. The highest *NN* values could be observed during sleep in the middle of the night at 0:00–2:30, whereas the lowest *NN* values occurred during the day at 15:00–19:00 in the awake phase.

Figure 7 demonstrates circadian rhythms in HRV parameters. The *NN* values for 12 h during the first half of the day (9:00–21:00) were consistently lower than those for 12 h during the second half of the day (21:00–9:00) (Figure 7A). While in the first third of the journey this difference was significant—evidencing the presence of the circadian rhythm—the significance progressively disappeared in the second and last thirds. This is because *NN* values raised with the ongoing journey for the first 12 h (9:00–21:00) while these for the next 12 h (21:00–9:00) did not change. The resulting circadian changes of *NN*, *SDNN*, and *RMSSD* over 24 h (Figure 7B) illustrated a decreasing *NN* from 350 ms/24 h for the first third down to 90 ms/24 h for the last third. The circadian changes of *SDNN* and *RMSSD* also decreased from the first to the last third but with a large overlap in the interquartile ranges.

## 4 Discussion

Endurance journeys with challenging long distances are becoming increasingly popular not only among athletes but also fitness enthusiasts eager to test their physical and psychological performance limits. Therefore, it is important to advance our knowledge about not only physiological but also psychological factors affecting the human body. These intense and prolonged factors may have negative consequences, mutually interact with each other, and determine the day-to-day performance, co-determining if such events will be successfully completed. The main purpose of this prospective study was to investigate insights and relationships between subjective and objective measures in a fitness enthusiast who completed an unsupported crossing of the North Atlantic.

### 4.1 Rower and boat data

Weight reduction after the transatlantic crossing was about 7% in our study, comparable to reported 5.5 kg (6.6%) (Keohane et al., 2019). Here a reduction in the percentage body fat can be expected due to the shift in muscle fuels from carbohydrates to fats (Keohane et al., 2019). Ultra-endurance races generally lead to reduced body mass and skeletal muscle mass (Johnson et al., 2016), also indicating also an increased risk for overuse injuries (Knechtle et al., 2009).

In the second half of the journey, the average boat speed (see Supplementary Figure S1) appears to follow proportionally the trend in the total sleep quality and mental wellbeing (Figures 2B, C). Even though 12 rowers were in the boat, this individual observation may indicate a positive relationship between the subjective perception and the boat performance; as well as indicate the effect of positive emotions on the performance in terms of coping strategies (Alschuler et al., 2020). Similarly, a poor progress in the yacht’s performance - even though due to issues outside the control (e.g., weather conditions)—was reported to cost disproportionally a lot of time and energy as well as emotional instability for skippers (Weston et al., 2009). The latter emotional instability was also confirmed by the monitored rower when her boat needed to parachute-anchor due to rough seas (March 27, see Supplement).

The progressive increase in ambient temperature (by about 22% from the first to the last third of the journey) and a reduction in its circadian variation (also by about 22%) during the journey may indicate an elevated thermal load on the rower, potentially contributing to her physical exhaustion. However, subjective scores on exhaustion and wellbeing do not directly confirm this expected influence (Figures 2B, C).

### 4.2 Subjective sleep quality

The total sleep quality is inversely influenced by wake ups during sleep and less by problems falling asleep, most likely due to a large physical exhaustion the temporal course of which inversely follows that of the sleep quality (Figures 2B, C). In analogy, sleep duration was positively related to vigor and inversely related to depression and fatigue (Pedlar et al., 2007). Since problems falling asleep did not correlate with the sleep quality and show up sporadically mainly in the first third of the journey, it could be hypothesized that the accumulated sleep dept was not a major factor in determining the sleep quality.

The initial drop of the sleep quality at the start of the journey can be expected due to an unusually high physical exhaustion with the body not yet adapted to the new 3 h rhythm of rowing and resting. Later, a prominent drop in the sleep quality occurs in the middle of the journey, overlapping with difficulties in having a continuous sleep and accumulated physical/mental tiredness; aggravated through the fact that a lot of rowing has already been completed, yet the finish is still out of sight. The last drop in the sleep quality resides close to the end, determined by a high physical exhaustion and frequent wake ups. The rower delivers her remaining performance while the finish line is still in a disappointing distance of a few days. In general, an inadequate recovery/sleep during the ultra-endurance rowing with high metabolic demands may offset the expected positive training effects of this rowing (Keohane et al., 2019).

Interestingly, the total sleep quality shows a rhythmicity of about 4 days in the first half of the journey, as also co-determined by the same rhythmicity of the wake up’s score. Problems falling asleep remain relatively low throughout the crossing and show only a few peaks in the first third of it, which is most likely due to accumulated tiredness and exhaustion easing falling asleep.

### 4.3 Subjective mental wellbeing

The total wellbeing shows a discontinuous course over the journey (Figure 2C), in line with the monitored positive emotions in an endurance athlete rowing across the North Atlantic (Alschuler et al., 2020). The mental wellbeing is inversely related to the physical exhaustion (Figure 2C). This is in contrast to the reported positive correlation between increased exertion and positive emotions in endurance athletes (Johnson et al., 2016), possibly because the monitored rower is a fitness enthusiast.

The mental wellbeing follows qualitatively the course of the total sleep quality with its aforementioned three drops over the course of the journey (Figure 2C). This is in line with the observation that mood disturbances arise from an imbalance between the physiological demand for sleep and the desire to complete the race as quickly as possible (Graham et al., 2012).

The middle of the monitored journey is especially critical as it shows a week-long prominent peak in tiredness (Figure 2C). Even more, this peak is aggravated farther by frequent wake ups and thus lowered sleep quality.

### 4.4 Objective HRV data

HRV parameters during exercise indicate a parasympathetic withdrawal and sympathetic augmentation, depending on the exercise intensity (Aubert et al., 2003), (Hammond and Froelicher, 1985). Correspondingly, HRV parameters associated with the parasympathetic modulation, namely, *NN, SDNN, RMSSD*, and *HF* increase from rowing to resting phases (Figures 2, 4) while the associated sympathovagal balance *LF/HF* decreases (Figure 6A). In fact, the extent of this increase reflects the modulation capacity of the autonomic body control or the body’s autonomic recovery while resting, which is vital for the regeneration of the body and the subsequent rowing performance.

Over the duration of the crossing, the autonomic recovery from exhausting rowing to short resting phases (including sleep) clearly decreases (Figure 2A), with the peak values (e.g., 160% for *RMSSD*) in the first week of the journey. Specifically, the increments in *NN, SDNN, RMSSD, HF* and the reduction in *LF/HF* from awake (incl. rowing) to sleep all weaken from 44.7% to 8%, 75.1% to 61.6%, 89.3% to 51%, 421.1% to 129.5%, and −31.8% to −1.5% respectively, from the first to the last third of the journey (Figure 6A).

In particular, *NN* values increase over the journey’s duration by about 20% (Figure 3), which confirms that an excessive exercise typically decreases heart rate at all workloads (Keohane et al., 2019). While this increase is significant during rowing, it is present but less pronounced during resting (Figure 5A). However, a closer look on awake and sleep phases shows that while *NN* in awake increases over the journey’s duration, *NN* in sleep decreases (Figure 6B). This results in a reduced awake-to-sleep variation in *NN* and thus indicates a worsened ability of the body’s autonomic modulation from awake to sleep and *vice versa*.

From the first to last third of the crossing, all *RMSSD, HF*, and *LF/HF* change respectively by about 16%, 65%, and 19% while *SDNN* experiences a temporary drop by about 14% in the second third (Figure 3). Consequently, the parasympathetic activity seems to increase during the journey while the sympathovagal balance is shifted towards the parasympathetic dominance. This aligns favorably with reports that an extreme exercise (like endurance running) leads to sympatholysis and rise in vagal tone (parasympathetic activity) in order to protect myocardial function from the very high circulating levels of neurotransmitters associated with adrenergic/sympathetic stimulation (Sztajzel et al., 2006).

The natural circadian 24 h rhythm of *NN* significantly decreases during the journey—a clear impact of the desynchronizing 3 h rowing and resting phases (Figure 7A). Interestingly, this rhythm seems to disappear at the cost of an increase in *NN* during the daytime (9:00–21:00) but not a potential decrease during the nighttime (21:00–9:00) (Figure 7A). Other HRV parameters also experience a decrease in their circadian rhythmicity but to a lesser extent (Figure 7B). This progressive loss of circadian rhythms may indicate disturbed endocrine and metabolic functions (Kaniusas, 2012).

### 4.5 Hypotheses

The main hypothesis of a decrease in the sleep quality and mental wellbeing over time is not confirmed; instead, a modulation with several ups and downs can be observed (Figures 2B, C). The hypothesis of a decreasing modulation capacity of the autonomic body control over time is confirmed (Figures 4, 6A).

The hypothesized similar changes in subjective and objective measures could not be observed. The temporal courses of the objective HRV parameters and their adaptation from rowing to resting do not appear to correlate with the courses of the subjective sleep quality and mental wellbeing (Figure 2). This dispersion may be due to a low temporal resolution of the implemented questionnaires. The hypothesis of a diminished circadian rhythm over time is confirmed (Figure 7A).

### 4.6 Limitations

This single case study does not allow generalization of results. However, the main purpose of the study was to learn—for the first time—about temporal individual evolutions of subjective and objective data and their interrelations during demanding ultra-rowing. A potential interference of measurements with intense daily activities of enthusiasts should be as little as possible, without putting any pressure. An ultra-rower can be hardly considered as a part of norm, which justifies data analysis at the intra-individual level.

Caffeine-based medication against seasickness in the first third of the journey and disrupted hormonal cycles throughout the whole row act as confounding factors for both subjective and objective measurements (especially for HRV). As a limitation, the body composition was not assessed before and after the crossing, nor were genetic profile and her personality traits, which act as further confounding factors. Although quantified amounts of raw ECG data and R peaks were lost during the ECG quality check, it is still highly rewarding to recognize that the periodically-rechargeable ECG recorder and the stored data within survived the 42 days’s journey on the high sea exposed to water, salt, and sweat. The variation in electrode positions has influenced ECG waveforms but not the derived HRV data. Pain was not monitored even though reported during the crossing. Proprietary simplified questionnaires were used to monitor the sleep, exhaustion, and mental wellbeing while minimizing interference with daily procedures. The use of validated and quite extensive questionnaires such as POMS (Profile Of Mood States) or EmRecQ (Emotional Recovery Questionnaire) is reported within extreme adventures (Johnson et al., 2016; Kenttä et al., 2006) and would certainly have increased the stability of data but would disadvantageously have increased the participant’s burden every second day. The baselines and follow-ups may have been influenced by pre-race tension and post-race fatigue (Parry et al., 2011); e.g., the Baseline 2 without rowing but with pre-race tension appears to be more stressful than the Baseline 1 with rowing but without pre-race tension (Figure 2A).

## 5 Conclusion

This paper addresses ultra-rowing outside of the competitive arena from a unique scientific perspective: subjective versus objective perception. We study human excellence under adverse conditions, aiming to provide fitness enthusiasts, athletes, support teams and organizers greater awareness as to the expected changes in the physiological and psychological wellbeing in long-duration, environmentally challenging activities.

The study results suggest recommendations for a better alignment of the rowing and resting phases throughout extended journeys (e.g., across oceans). These recommendations should allow for both sufficient performance and wellbeing, similarly to athletes and their coaches aiming to balance training and recovery when striving for optimal performance without staleness. Our recommendations are: 1) successive but not concurrent introduction of the exceptionally exhausting rowing and unusually short relaxation periods at the beginning of the journey in order to increase progressively but not abruptly the total psychophysical load over time (e.g., through a temporary introduction of 6 h rhythm of rowing and resting); 2) additional recreational/mental/food/family support in the middle of the journey to counteract there the expected peak in the perceived tiredness and downs in sleep and mental wellbeing scores.

The gained insights provide a basis for more effective preparation for undertakings with extreme physical exhaustion, disregarding natural circadian sleep cycles. This may lead to more favorable emotional, behavioral, and physiological responses of participating individuals.

## Data Availability

The raw data supporting the conclusion of this article will be made available by the authors on request, without undue reservation.
