# Supplementary material for: Mental and physiological wellbeing while rowing across the North Atlantic: a single-case study of subjective versus objective data
Source: Front Physiol. 2023 Sep 19;14:1244438. doi: 10.3389/fphys.2023.1244438 (PMC10546170; doi:10.3389/fphys.2023.1244438)
Supplement: Supplementary file 1 [file Presentation1.pdf]

frontiers

Supplementary Material

Mental and physiological wellbeing while rowing across the North Atlantic: a single-case study of subjective versus objective data

Klaus Zeiner, Babak Dabiri, Ciara Burns, Lena Kummer, Eugenijus Kaniusas*

*** Correspondence:** Univ. Prof. Eugenijus Kaniusas: kaniusas@tuwien.ac.at

1 Supplementary Figures and Tables

1.1 Supplementary Figures

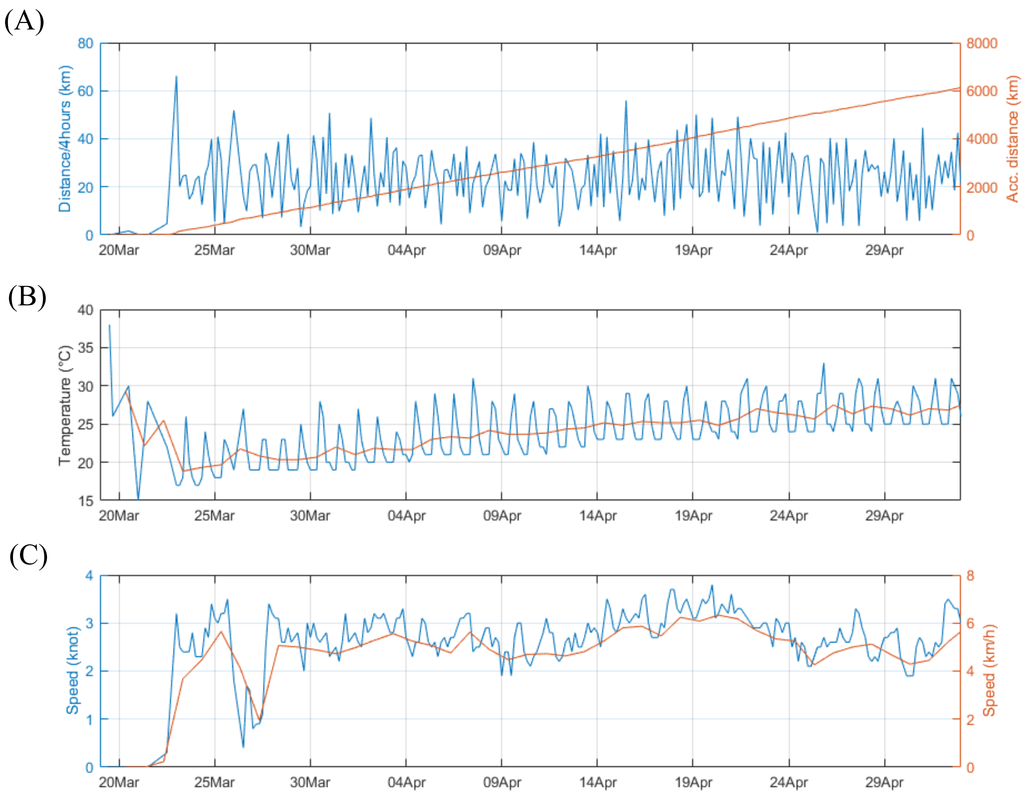

Supplementary Figure 1. Recorded boat data. (A) Distance traveled per 4 hours (blue) versus the accumulated distance (red). (B) Outside temperature (blue) with its daily average (red). (C) The speed (blue) with its daily average (red).

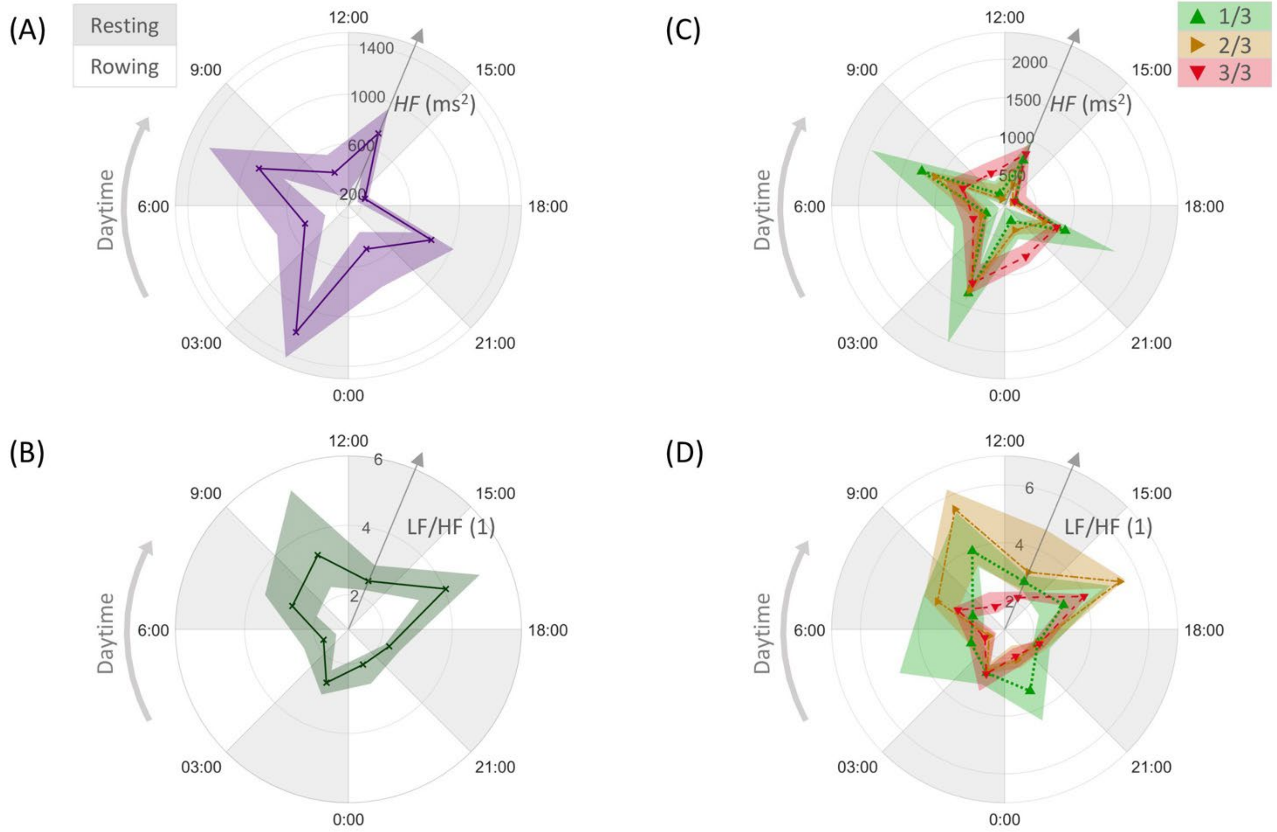

Supplementary Figure 2: HRV parameters for rowing phases (white sectors) and resting phases (grey sectors) over 24 hours. The median and interquartile range (25 and 75%) are shown for the medians of the 5min interval values within each phase. (A) The values of *HF* and (B) *LF/HF* for the whole journey. (C) The values of *HF* and (D) *LF/HF* within the first, second, and the final third of the journey, coded with green, yellow and red colors, respectively.

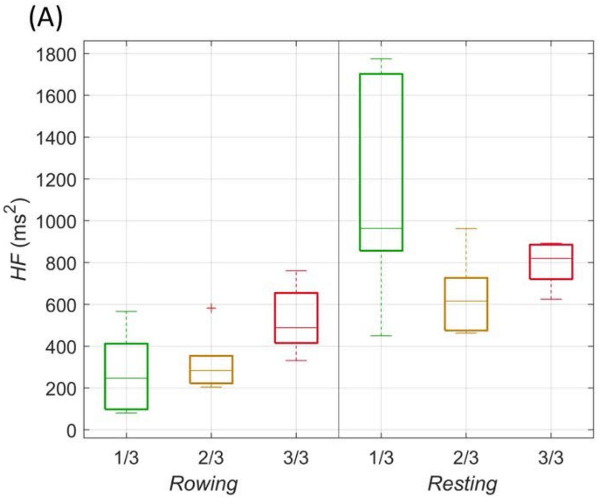

Supplementary Figure 3: HRV parameters for 3 hour rowing phases and 3 hour resting phases within the first, second, and the final third of the journey, coded with green, yellow and red colors, respectively. (A) The distribution of quartiles of the 5min interval values of *HF* and (B) *LF/HF*. Dashed lines indicate whiskers extending to the most extreme data points.
